# Seq-SymRF: a random forest model predicts potential miRNA-disease associations based on information of sequences and clinical symptoms

**DOI:** 10.1038/s41598-020-75005-9

**Published:** 2020-10-21

**Authors:** Jinlong Li, Xingyu Chen, Qixing Huang, Yang Wang, Yun Xie, Zong Dai, Xiaoyong Zou, Zhanchao Li

**Affiliations:** 1 School of Chemistry and Chemical Engineering, Guangdong Pharmaceutical University, Guangzhou, 510006 People’s Republic of China; 2 School of Chemistry, Sun Yat-Sen University, Guangzhou, 510275 People’s Republic of China; 3 Key Laboratory of Digital Quality Evaluation of Chinese Materia Medica of State Administration of Traditional Chinese Medicine, Guangzhou, 510006 People’s Republic of China

**Keywords:** Diseases, Machine learning, Computational biology and bioinformatics, Classification and taxonomy

## Abstract

Increasing evidence indicates that miRNAs play a vital role in biological processes and are closely related to various human diseases. Research on miRNA-disease associations is helpful not only for disease prevention, diagnosis and treatment, but also for new drug identification and lead compound discovery. A novel sequence- and symptom-based random forest algorithm model (Seq-SymRF) was developed to identify potential associations between miRNA and disease. Features derived from sequence information and clinical symptoms were utilized to characterize miRNA and disease, respectively. Moreover, the clustering method by calculating the Euclidean distance was adopted to construct reliable negative samples. Based on the fivefold cross-validation, Seq-SymRF achieved the accuracy of 98.00%, specificity of 99.43%, sensitivity of 96.58%, precision of 99.40% and Matthews correlation coefficient of 0.9604, respectively. The areas under the receiver operating characteristic curve and precision recall curve were 0.9967 and 0.9975, respectively. Additionally, case studies were implemented with leukemia, breast neoplasms and hsa-mir-21. Most of the top-25 predicted disease-related miRNAs (19/25 for leukemia; 20/25 for breast neoplasms) and 15 of top-25 predicted miRNA-related diseases were verified by literature and dbDEMC database. It is anticipated that Seq-SymRF could be regarded as a powerful high-throughput virtual screening tool for drug research and development. All source codes can be downloaded from https://github.com/LeeKamlong/Seq-SymRF.

## Introduction

MiRNAs are a class of non-coding RNA with a length of about 22 nucleotides, which affect biological processes by regulating gene expression. It binds to the 3′ untranslated region (UTR) of the target mRNA through sequence-specific base pairing to inhibit the expression of the target mRNA^1^. So far, various biological experiments have verified that miRNAs play a vital role in many significant biologic processes^2^ and are related to a wide range of human diseases, such as cancer, immune-related diseases, Parkinson’s disease and Alzheimer’s disease^3–6^. Therefore, the identification of associations between miRNAs and diseases is useful to understand the pathogenic mechanisms and contributes to the development of personalized treatment. However, the traditional experimental methods are undoubtedly time-consuming and expensive because of a large number of miRNAs and the complexity of disease types.

With rapid accumulation and development of various biological information databases, identifying miRNA-disease associations through computational approaches attracts widespread attention from scientific communities. Various theoretical methods have been proposed and utilized to identify the associations. These existing approaches can be roughly divided into two categories: (1) machine learning-based method^7–13^, (2) score-based method^14–19^. In the first category, support vector machine, neural network, deep convolutional neural network and logistic regression are usually used to build models based on different features including topology feature, miRNA and disease similarity feature. In the second category, some scoring strategies or methods are employed based on various similarities among miRNAs and diseases.

Although these existing methods exhibited superior prediction accuracy, there are still some improvements in the recognition of miRNAs-disease associations. For example, most methods employed miRNA functional similarity matrix calculated by Wang^20^ in 2010 to characterize miRNAs. Owing to the rapid accumulation of newly discovered miRNAs, however, the functional similarity matrix is outdated and needs to be recalculated. Additionally, similarity score-based characterization methods are severely limited by information from other miRNAs. In fact, each miRNA has its unique sequence, so it is more reliable to describe each miRNA as a feature vector by mining base sequence properties. In addition, the use of semantic similarity based on MeSH (Medical Subject Headings) to characterize the disease also encountered the same problem. For some new diseases, their directed acyclic graph is not described to calculate semantic similarity because their affiliation and pathogenesis are unclear. As a matter of fact, the clinical symptom is one of the most intuitive features for disease. Community health professionals and general practitioners derive most of their knowledge of the symptoms of individual diseases from hospital-based observations^21^. Therefore, clinical symptom information can be utilized to characterize disease and calculate similarity. Moreover, almost all the exsting methods treat randomly generated unknown associations as negative samiples because there is no database dedicated to collecting non-associated pairs between miRNAs and diseases. Obviously, those negative samples generated by random matching must contain some potential positive samples, which affects the prediction performance of the model. Therefore, it is necessary to develop a strategy to select reliable negative samples.

For the reasons mentioned above, a novel computational method was proposed to identify potential miRNA-disease associations. In this approach, miRNAs and diseases were characterized by features derived from the sequence information and clinical symptoms, respectively. Negative examples were obtained through cluster analysis based on Euclidean distance. The random forest algorithm was utilized to construct model. The prediction and practical application performance of the current method was evaluated by using the fivefold cross-validation and some case studies, respectively.

## Materials and methods

### MiRNA characterization

Different from existing methods, the sequence information was used to characterize miRNAs. Firstly, we retrieved 1917 pre-miRNA sequence information from the miRBase^22^ database (Release 22.1, hairpin.fa). Secondly, the miRNA sequence secondary structure with dot-bracket notation and minimum free energy was calculated and obtained through the RNAfold^23^. In the dot-bracket notation, "(" and ")" represent the paired nucleotide local near in the 5-end and 3′-end, respectively. The dot "." represents the unpaired one. Thirdly, the 10 global and 32 local features were calculated. The global features contain symmetric difference, number of base pairs, GC content, length base pair ratio (length of the sequence/the number of base pairs), sequence length, length of central loop, free energy per nucleotide, bulge number, tail length and the number of tail(s). The local features mean 8 possible structure compositions such as "(((", "((.", "(..", "(.(", ".((", ".(.", "..(" and "…" for any three adjacent nucleotides. There are 4 kinds of bases (A, U, C, G) in the middle position to constitute 32 different combinations (8 × 4). In addition, the statistical values of 84 nucleotides (4 base count, 16 dimer count and 64 codon count) were also calculated. Finally, a feature vector with 126 (10 + 32 + 84) dimensions containing global, local and nucleotide statistics count was employed to characterize miRNA sequence.

### Disease characterization

Symptoms are abnormal states that occur when the body has a lesion. In the daily treatment of the hospital, through a comprehensive analysis of the symptoms, the doctor can make a preliminary understanding of the patient's state and determine further treatment options. Thus, clinical symptoms were utilized to characterize the disease. The associations between disease and their clinical symptoms were retrieved from the human symptom-disease network^24^, in which association scores were calculated by using the term frequency-inverse document frequency algorithm and term frequency was replaced by the absolute co-occurrence. Finally, each of the diseases can be characterized through a feature vector with 322 dimensions, in which every element corresponding to one specific symptom and was encoded as a value larger than or equal to zero for explaining the association strength between disease and symptom.

### Human miRNA-disease associations characterization

In this study, the human miRNA-disease associations data were downloaded from the HMDD database (v3.0)^25^. A total of 18,733 experimentally confirmed associations between 1208 miRNAs and 894 diseases were obtained. After removing some associations, in which miRNA and disease were not involved in the miRBase database and the symptom-disease database, a benchmark data set containing 7456 associations between 917 miRNAs and 339 diseases was finally obtained. A feature vector with 448 (126 + 322) dimensions by concatenation of the miRNA feature and disease feature was utilized to characterize miRNA-disease association pair.

### Benchmark data set redundancy analysis

To evaluate the redundancy of the benchmark data set, we calculated the similarity of any two miRNAs, two diseases and two miRNA-disease association pairs, respectively. For two miRNAs, we performed sequence alignment to calculate similarity by using CD-HIT software^26^. For two diseases, the Jaccard coefficient is calculated to evaluate similarity based on the disease symptoms feature vector. For two miRNA-disease association pairs m(*i*)-d(*p*) and m(*j*)-d(*q*), their similarity (mDPS) can be calculated according to the Eq. (1):

1 $$mDPS = \frac{{Sm\left( {i, \, j} \right) + Sd\left( {p, \, q} \right)}}{2}$$

where, *Sm*(*i*,*j*) and *Sd*(*p*,*q*) mean the similarity of two miRNAs m(*i*) and m(*j*) as well as two diseases d(*p*) and d(*q*).

### Negative sample selection

Identification of miRNA-disease associations is a binary classification problem, i.e. infer whether a miRNA is associated with a disease. However, there is no database collecting negative associations. Therefore, we treated our method as positive-unlabeled learning and select the reliable negative sample from the unlabeled sample (i.e. un-included in the HMDD database). All confirmed experiment associations were considered as positive set (i.e. the constructed benchmark data set), and the remaining 303,407 (917  × 339 – 7456) associations were regarded as the unlabeled set. We further selected the reliable negative sample from the unlabeled set with following steps: (1) Calculate mean of all the samples in the positive set with each dimension to form a standard 448 dimensions vector as the cluster center. (2) Calculate the Euclidean distance between each sample in unlabeled set and cluster center. (3) Calculate the average Euclidean distance (AED). (4) Treat the sample in unlabeled set as a reliable negative sample if its Euclidean distance is higher than AED. (5) Generate a reliable negative sample data set.

### Model construction and evaluation

**Figure 1:**
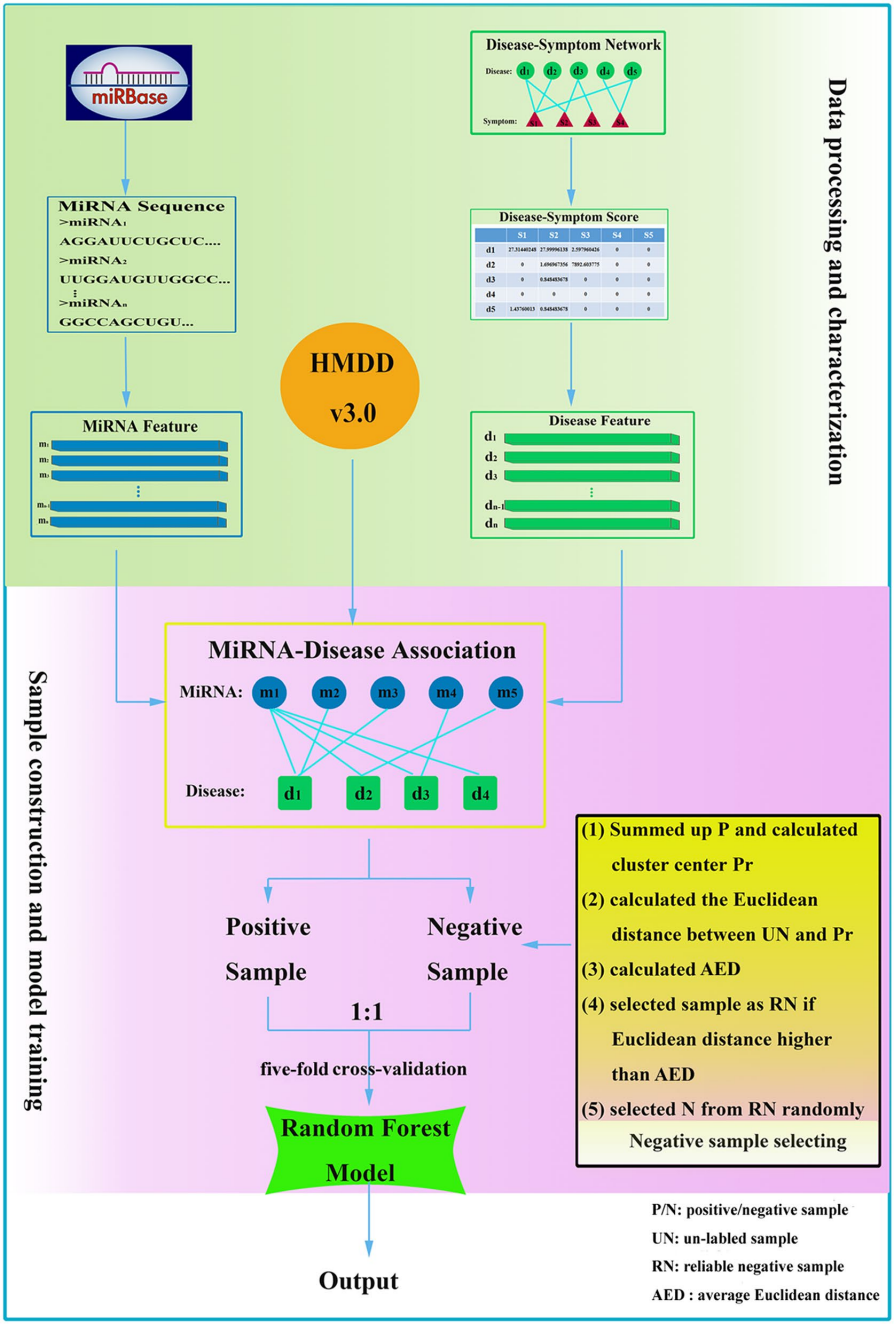
Flowchart of the current method.

In this study, we constructed a random forest classifier model to predict the potential miRNA-disease associations. Firstly, the information of miRNA sequences, disease-symptom associations and miRNA-disease associations were retrieved from miRBase database, disease-symptom database and HMDD database (v3.0), respectively. Secondly, the characterization of each miRNA-disease association was constructed with a 448 dimensions feature. Then, a training data set was formed with the ratio 1:1 of positive and negative samples. Finally, the random forest algorithm was utilized to construct model for identifying potential miRNA-disease associations. Figure 1 depicts the flowchart of our proposed framework.

To evaluate the identification performance of the model, fivefold cross-validation was utilized based on the benchmark data set, which was divided into 5 parts, the 4 parts was treated as training set and the other 1 part was treated as test set in turn. We repeated 10 times to obtain the statistic results, including accuracy (Acc), specificity (Spe), sensitivity (Sen), precision (Pre), Matthews correlation coefficient (Mcc), the areas under receiver operating characteristic curve (AUROC) and precision recall curve (AUPRC). The Acc, Spe, Sen, Pre and Mcc can be calculated according to Eqs. (2)–(6):

2 $${\text{Acc}} = \frac{TP + TN}{{TP + TN + FP + FN}} \times 100\%$$

3 $${\text{Spe}} = \frac{TN}{{TN + FP}} \times 100\%$$

4 $${\text{Sen}} = \frac{TP}{{TP + FN}} \times 100\%$$

5 $$\text{Pre} = \frac{TP}{{TP + FP}} \times 100\%$$

6 $${\text{Mcc}} = \frac{TP \times TN - FP \times FN}{{\sqrt {(TP + FN) \times (TN + FN) \times (TP + FP) \times (TN + FP)} }}$$

where, TP, TN, FP and FN represent the number of true positive, the number of true negative, the number of false positive and the number of false negative, respectively. Receiver operating characteristic curve (ROC) takes true positive rate (sensitivity) as the vertical coordinate and false positive rate (1-specificity) as the horizontal coordinate. Also, Precision-recall curve (PRC) takes precision as the vertical coordinate and recall as the horizontal coordinate. The higher AUROC and AUPRC, the better performance of the model.

## Results

### Redundancy of the benchmark data set

**Figure 2:**
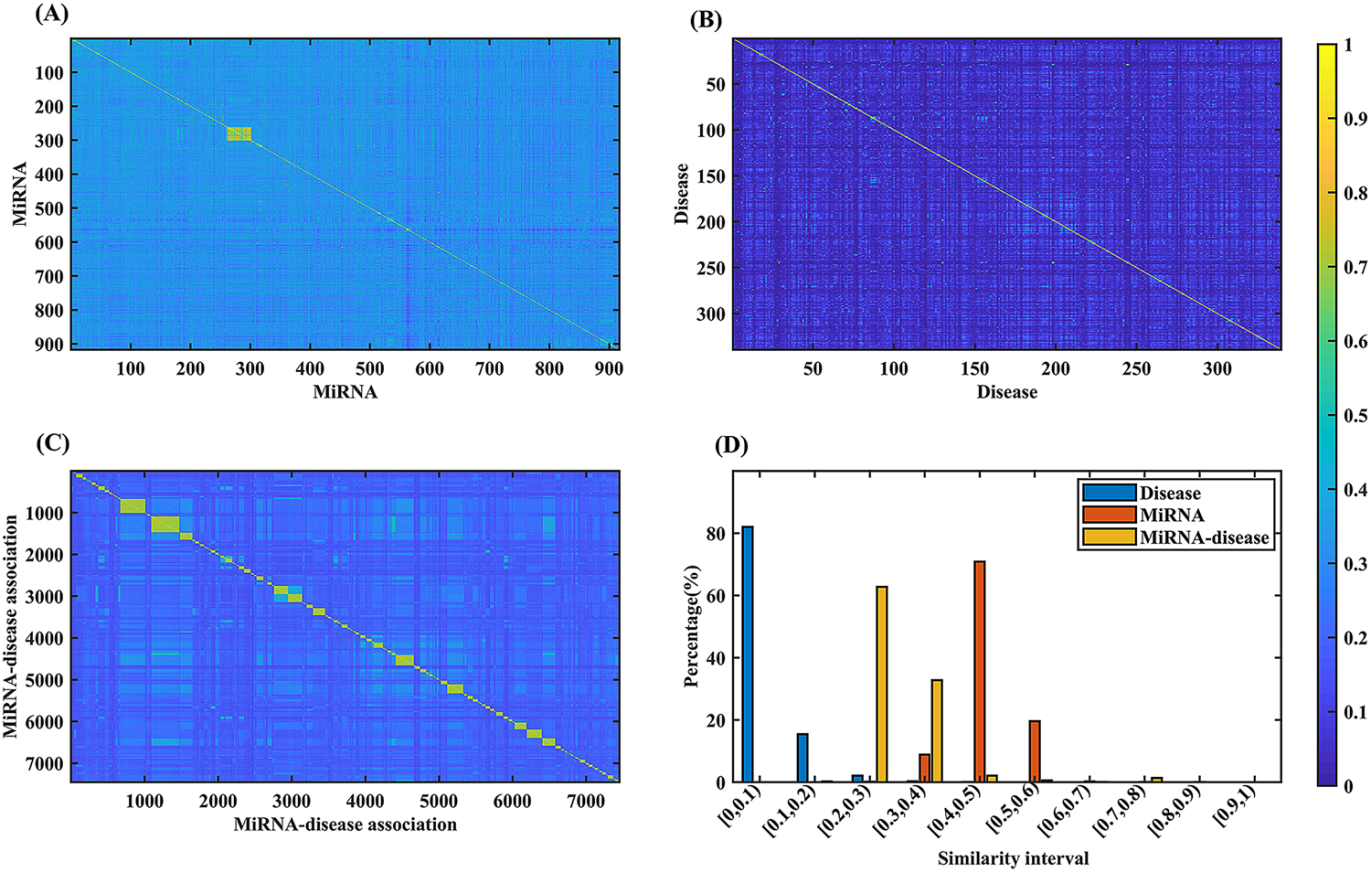
The similarity and statistical results of the benchmark data set. (**A**–**C**) describe the similarity of any two miRNAs, two diseases and two miRNA-disease associations, respectively. (**D**) Describes the distribution of miRNA, disease and miRNA-disease association similarity values.

Similarity values of any two miRNAs, two diseases and two miRNA-disease association pairs as well as their statistical results were shown in Fig. 2A–D, respectively. For miRNAs, their similarity values mainly are concentrated in [0.4, 0.5) (i.e. value higher than or equal to 0.4 and lower than 0.5, the same below), accounting for 70.88%. The proportions of interval [0.2, 0.3), [0.3, 0.4), [0.5, 0.6), [0.6, 0.7), [0.7, 0.8), [0.8, 0.9) and [0.9, 1.0] are 0.13%, 8.90%, 19.74%, 0.22%, 0.13%, 0.0754% and 0.0076%, respectively. Similarity values of any two miRNAs are not included in the range of [0, 0.1) and [0.1, 0.2). The similarity values of any two diseases are always lower than 0.8. The 81.98% and 15.45% of all similarity values are located in the range of [0, 0.1) and [0.1, 0.2), respectively. Only 2.11%, 0.32%, 0.0698%, 0.0471%, 0.0070% and 0.0087% concentrated in the range of [0.2, 0.3), [0.3, 0.4), [0.4, 0.5), [0.5, 0.6), [0.6, 0.7) and [0.7, 0.8). And, distribution of miRNA-disease associations similarity values is more dispersed. The 62.75% and 32.76% of similarity values are concentrated in the range of [0.2, 0.3) and [0.3, 0.4). The 0.27%, 2.15%, 0.55%, 0.0969%, 1.41%, 0.0135% and 0.0017% are located in the of [0.1, 0.2), [0.4, 0.5), [0.5, 0.6), [0.6, 0.7), [0.7, 0.8), [0.8, 0.9) and [0.9, 1.0], respectively. These results indicate that the constructed benchmark data set has a low redundancy.

### Optimization of model parameter

Random forest as a decision tree-based algorithm, the number of decision tree and predictor variable at each decision split have an important impact on prediction performance. Therefore, the prediction performance of the constructed model was optimized based on the grid search strategy, in which change the tree number from 100 to 1000 with interval 100 and feature number from 2^1^, 2^2^, …, to 2^5^. In addition, a default value (i.e. square root of the total variable number) was also used. The Acc, Sen, Spe, Pre, Mcc, as well as AUROC and AUPRC derived from each combination of tree number and variable number were listed in Supplement Tables 1–7.

From the results, we can observe that the number of trees has less impact on model performance. When the number of trees is determined, the model performance enhance with the increase of the number of selected variables. And, the performance remains stable when the number of selected variables is greater than the default value. Moreover, it takes more time to train the model when the numbers of trees and selected variables are larger. Therefore, the optimal model was constructed by setting 100 and 22 for the number of trees and selection features. Finally, the optimized model achieved the average Acc of 92.27%, Spe of 92.93%, Sen of 91.62%, Pre of 92.85%, and Mcc of 0.8456 and the corresponding AUROC and AUPRC were 0.9756 and 0.9787, respectively.

### Performance comparing of different negative examples selection strategy

**Figure 3:**
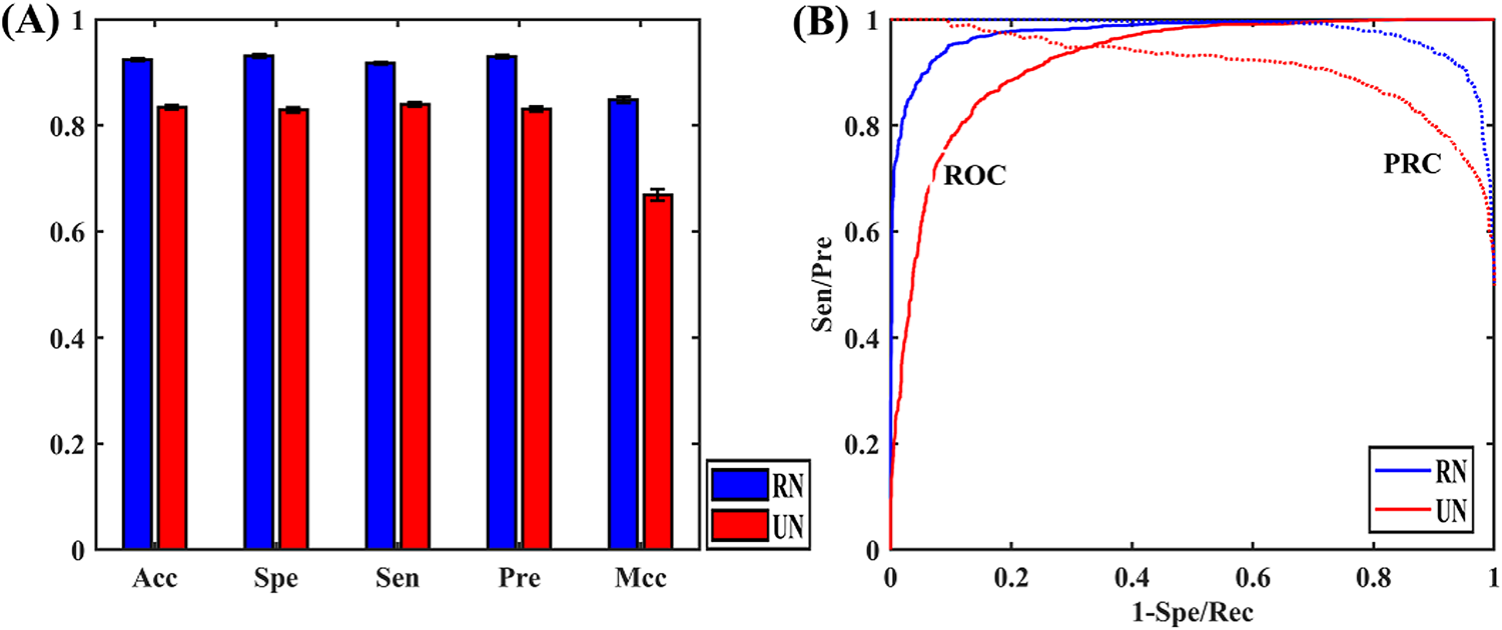
Comparison of different negative example selection strategies. (**A**) Describes the comparison of prediction results between two selection negative methods, and (**B**) describes the ROC and PRC for different selection strategy. In this figure, selecting negative sample from reliable negative sample (RN) was represented in blue and selecting negative sample from unlabeled negative sample (UN) was represented in red.

In order to demonstrate the reliability of the current negative sample selection strategy, we compared it with the random selection strategy from the unlabeled set. The result of the two selection strategies was shown in Fig. 3. When the negative sample was randomly selected, Acc, Spe, Sen, Pre, Mcc, AUROC, and AUPRC was 83.67%, 82.93%, 84.40%, 83.17%, 0.6733, 0.9184 and 0.9072, respectively. It is 8.60%, 10.00%, 7.22%, 9.68%, 17.23%, 5.72%, 7.15% lower than that of the current method, respectively. Further, we calculated the relative standard deviation (RSD). For the current method, the RSD% of Acc, Spe, Sen, Pre and Mcc is 0.28, 0.31, 0.24, 0.32 and 0.60, respectively. These values are always lower than those of the random selection strategy (0.43%, 0.53%, 0.41%, 0.48% and 1.08%). The results demonstrate that the selection of negative sample is effective and reliable. The lower RSD% also proves that the current selection strategy can make the model more robust to negative samples.

### Reliability of negative sample

**Figure 4:**
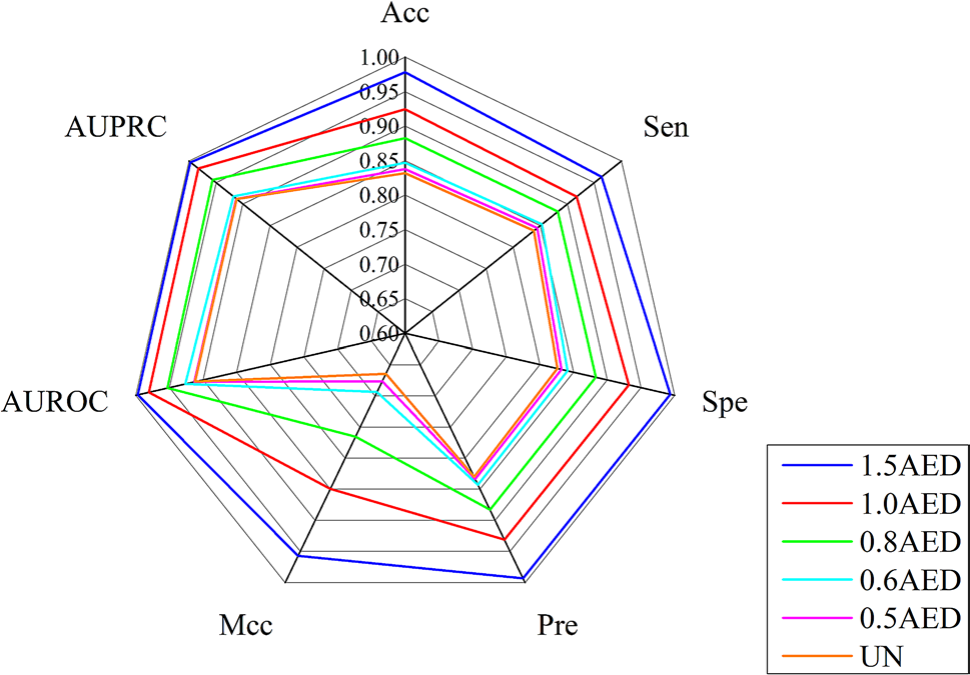
The comparison of 7 evaluation metrics among different thresholds.

We further construct five reliable negative sample data sets based on the different AED threshold (1.5AED, 1.0AED, 0.8AED, 0.6AED and 0.5AED) to investigate the impact on the performance of the model (nAED represents n × AED). The results of different thresholds were shown in Fig. 4. The performance of the model decreases gradually with the decrease of the threshold. When the threshold is lower than 0.5AED, there is no difference for the current and random negative samples selection strategy. Therefore, we can conclude that the reliability of negative samples has a positive impact on the prediction performance. Finally, the 1.5AED was chosen as a threshold to select the reliable negative sample.

### Proportion of positive and negative samples

**Figure 5:**
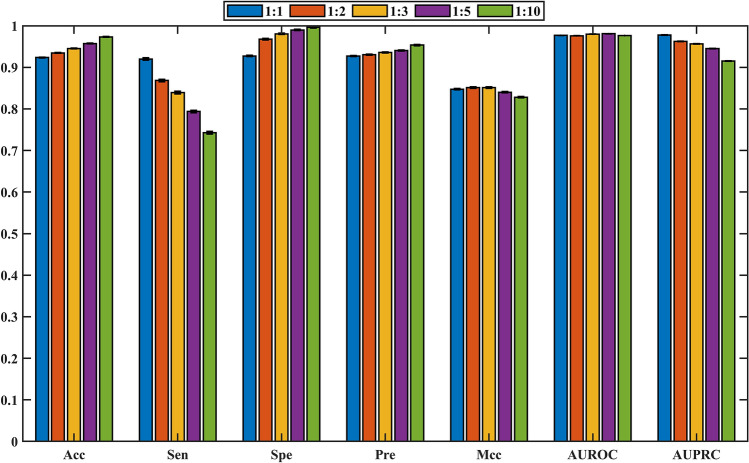
The comparison results among different ratios of positive and negative samples.

In machine learning, the ratio between positive samples and negative samples also affects the model performance. To explore this effect, five training data sets with different rations (1:1, 1:2, 1:3, 1:5 and 1:10) were constructed. Results of the fivefold cross-validation test were shown in Fig. 5 (Please note that in order to ensure sufficient negative samples, the selection threshold of reliable negative samples is set to 1.0AED). As the number of negative samples increases, Acc, Spe and Pre increased slowly, Mcc and AUROC fluctuated slowly and randomly, AUPRC decreased continuously. But the Sen decreased significantly with the ratio increased from 1:1 to 1:10. Because the purpose of current research is to identify potential positive samples (i.e. model should have a higher sensitivity), the optimal ratio of positive and negative samples was set to 1:1.

### Analysis of feature importance

The SHAP (Shapley additive explanation) value was originally developed to estimate the importance of individual player in a collaborative team. It aims to distribute revenue among players based on their relative importance to final outcome of game^27,28^. Based on this theory, SHAP value has been applied to machine learning model for evaluating feature importance. In this study, a random forest model was constructed and the SHAP value was calculated for each feature. The top 50 important features were analyzed, such as 29 miRNAs features and 21 disease symptoms, and the results were shown in the supplementary Fig. 1. Among these 29 miRNAs features, there were 10 global features of miRNAs secondary structure, 4 three adjacent paired, 4 unpaired nucleotides for each base, and 4 base composition of miRNAs sequence. Similarly, among 21 disease symptoms, we found common symptoms of "vomiting", "nausea", "weight loss", "Asthenia", etc., which are often accompanied by different diseases. We can conclude that the information of miRNAs sequence and disease symptom can be used as features to identify miRNA-disease association.

### Prediction capability for new miRNA

Identifying potential miRNAs associated with known diseases plays an important role in drug target discovery and new drug research. To further verify the prediction capability of our method for disease-related potential miRNAs, we removed the miRNA from our data set if its similarity is higher than a specific threshold. Here, four non-redundant data sets were constructed based on different thresholds: 0.9, 0.8, 0.7 and 0.6 (When the threshold is lower than 0.6, the number of miRNAs is too less to have its statistical significance). Results of the fivefold cross-validation based on the various non-redundant data sets were listed in Table 1. The AUROC and AUPRC remain stable at about 0.9962 and 0.9970. When the threshold is higher than 0.6, Acc, Sen, Spe, Pre and Mcc have a very narrow fluctuation range (< 1%). In summary, these results reveal that our method is insensitive for the redundancy of data set, and has an outstanding performance for identifying disease-associated miRNA.

**Table 1:** The results of fivefold cross-validation test from the different non-redundant data sets.

Threshold	Acc (%)	Sen (%)	Spe (%)	Pre (%)	Mcc	AUROC	AUPRC
0.9	97.98	96.34	99.61	99.60	0.9601	0.9968	0.9974
0.8	97.87	96.42	99.32	99.30	0.9578	0.9955	0.9965
0.7	97.39	95.47	99.31	99.29	0.9486	0.9973	0.9978
0.6	97.57	95.35	99.80	99.80	0.9524	0.9950	0.9964

### Identification ability for new diseases

Identification of potential diseases associated with known miRNAs contributes to the study of pathological mechanism. Similarly, we remove the disease from our data set if its similarity value is greater than the threshold: 0.7, 0.6, 0.5, 0.4, 0.3 and 0.2, respectively. Please note that the highest similarity score is 0.7370 in our data set, and only 290 positive samples remain when the threshold is set to 0.1. Finally, we constructed six non-redundant data sets, and the corresponding results were shown in Table 2. As the threshold decreases from 0.7 to 0.2, all indicators change slightly around a certain value. For example, Acc and Sen fluctuate slowly around an average of 97.81% and 96.34%. Even if the threshold is reduced to 0.2, our method still achieves Spe of 98.68%, Pre of 98.61%, Mcc of 0.9469, AUROC of 0.9960 and AUPRC of 0.9960. These results demonstrate that our method can identify potential miRNA-related diseases.

**Table 2:** The results of different non-redundant data sets.

Threshold	Acc (%)	Sen (%)	Spe (%)	Pre (%)	Mcc	AUROC	AUPRC
0.7	97.89	96.40	99.37	99.35	0.9582	0.9958	0.9967
0.6	98.03	96.51	99.55	99.54	0.9609	0.9955	0.9963
0.5	97.97	96.52	99.43	99.41	0.9598	0.9974	0.9979
0.4	97.90	96.41	99.40	99.38	0.9550	0.9962	0.9975
0.3	97.73	96.13	99.31	99.30	0.9550	0.9977	0.9981
0.2	97.33	96.04	98.68	98.61	0.9469	0.9934	0.9942

### Prediction capability for potential miRNA-disease associations

To further verify the robustness of our method, we construct a series of non-redundant data sets according to the different thresholds following these steps: (1) Set a threshold. (2) Randomly selected a positive association and calculate its similarity with other positive associations. (3) Removed the selected positive association if its similarity value is higher than the specific threshold, otherwise, keep it in the positive sample set. (4) Repeated step 2, until the similarity value of any two positive association pairs is lower than the threshold, and the obtained set was called as non-redundant positive sample set. (5) Randomly choose an association from reliable negative samples, and calculate its similarity values with each association in the positive sample set as well as in the negative sample set. (6) Deleted the chosen association from the reliable negative samples if similarity values are higher than the threshold, otherwise, remain it. The obtained set is known as non-redundant negative sample set. (7) Repeated step 5, until the size of the non-redundant positive sample set equals to non-redundant negative sample set. (8) Combined the non-redundant positive sample set with the non-redundant negative sample set to construct the non-redundant training data set. Finally, we set two thresholds 0.9 and 0.8 to build the two non-redundant training data sets (only 53 samples contained in the non-redundant negative sample set when the threshold is set as 0.7, it is unable to construct the non-redundant training data set with positive–negative ratio 1:1). Supplementary table 8 introduces the process of this section. The results of the fivefold cross-validation were shown in Table 3. The Acc of 97.88%, Sen of 96.44%, Spe of 99.33%, Pre of 99.31%, Mcc of 0.9581, AUROC of 0.9964 and AUPRC of 0.9973 are obtained when the threshold is set to 0.9. The Acc, Sen, Spe, Pre, Mcc, AUROC and AUPRC only decrease 0.29%, 0.52%, 0.06%, 0.07%, 0.0057, 0.0023 and 0.0015, when change the threshold from 0.9 to 0.8. There results demonstrate that the current method has a prominent robustness and can identify potential miRNA-disease associations.

**Table 3:** The results of fivefold cross-validation based on the two non-redundant miRNA-disease associations data sets.

Threshold	Acc (%)	Sen (%)	Spe (%)	Pre (%)	Mcc	AUROC	AUPRC
0.9	97.88	96.44	99.33	99.31	0.9581	0.9964	0.9973
0.8	97.59	95.92	99.27	99.24	0.9524	0.9941	0.9958

### Independent test

In this section, two methods were used to further verify the performance of our method. In the first method, we randomly divided the 7456 positive samples obtained from HMDD 3.0 into two parts, of which 5965 samples (80%) and 1491 samples (20%) were considered as training data set and independent data set. In the second method, 4119 positive samples retrieved from HMDD 2.0 were considered as training data set, and 2640 newly known miRNA-disease associations in the HMDD 3.0 were treated as the independent data set. All samples in the independent test data set were excluded in the training data set. To perform a truly and strict blind test, the positive sample was divided into five parts, one was treat as test set and other four parts were treated as training set. Also, all negative samples were constructed in each fold based on the subset of positives samples. For the first method, fivefold cross-validated obtained an average Acc of 96.07%, Sen of 96.23%, Spe of 95.91%, Pre of 95.97%, Mcc of 0.9216, AUROC of 0.9928 and AUPRC of 0.9945 based on the training data set. For the second method, fivefold cross-validated acquired an average Acc of 92.32%, Sen of 92.13%, Spe of 92.50%, Pre of 92.74%, Mcc of 84.79%, AUROC of 0.9828% and AUPRC of 0.9870 based on the training data set. Further, two training data sets were employed to train model and the corresponding two independent test data sets were adopted to evaluate prediction performance. The 1433 from 1491 samples and 2555 from 2640 samples were correctly identified with an Acc of 96.11% and Acc of 96.78%, respectively. These results showed that our method is reliable to predict the potential miRNA-disease association.

### Comparison with other methods

**Figure 6:**
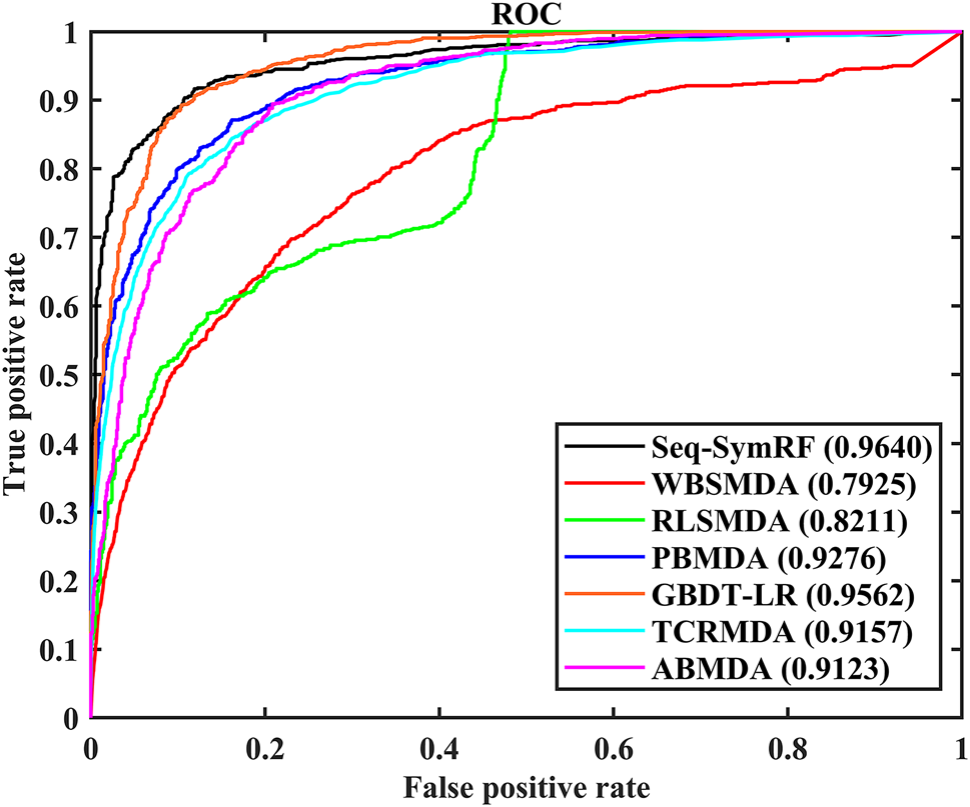
Performance comparisons between Seq-SymRF and WBSMDA, RLSMDA, PBMDA, GBDT-LR, TCRMDA as well as ABMDA.

Base on fivefold cross-validation and a data set including 4119 known associations between 415 miRNAs and 327 diseases from HMDD (v2.0), the proposed Seq-SymRF is further evaluated and confirmed by comparing with the six methods of WBSMDA^14^, RLSMDA^8^, PBMDA^15^, GBDT-LR^12^, TCRMDA^16^ and ABMDA^13^. The ROCs of Seq-SymRF and other methods are shown in Fig. 6. The AUROC of Seq-SymRF is 0.9828, and those of WBSMDA, RLSMDA, PBMDA, GBDT-LR, TCRMDA, and ABMDA is 0.7925, 0.8211, 0.9276, 0.9562, 0.9157 and 0.9123 respectively. In conclusion, the current method demonstrates good prediction performance and can be used to identify potential miRNA-disease associations.

### Case study

To demonstrate the application ability of our method, we implement the following two case studies: (1) Removed two common diseases of breast neoplasms and leukemia from our data set and predicted their association score with each miRNA. (2) Deleted hsa-mir-21 from our data set and predicted its association score with each disease. The top 50 results are validated by dbDEMC^29^ or the literature.

Breast cancer is one of the most fatal diseases among females. According to the global cancer statistics 2018^30^, female breast cancer is the second most common cancer, accounting for 18.6% of cancer deaths, behind lung cancer. MiRNA has a practical effect on the treatment and diagnosis of breast cancer. For example, miRNA-223 can maintain cell proliferation of breast cancer cells through targeting FOXO1^31^ and let-7a can inhibit growth and migration of breast cancers cell by targeting HMGA1^32^. Taking breast neoplasms as case study, our random forest model was implemented to prioritize candidate miRNAs. Supplementary table 9 lists the prediction score with different miRNA. From the table, there are 33 of top 50 potential related miRNAs were confirmed to be associated with breast neoplasms based on the dbDEMC database.

Leukemia is a group of life-threatening malignant disorders of the blood and bone marrow. It is a dangerous disease that may occur at all ages, from the newborn to the very old^33^. As a special biomarker, miRNA also has a significant impression in leukemia treatment. The identification of the potential association between miRNA and leukemia is very helpful in the development of therapeutic drugs. The prediction score between leukemia and each candidate miRNA is listed in Supplementary table 10. In our model, 36 of the top 50 potential related miRNAs were confirmed based on the dbDEMC database.

Mir-21 is a well know miRNA, and it has been proved to be associated with many diseases, especially cancer. Its association score with various diseases is predicted and listed in Supplementary table 11. There are 32 of top 50 potential associations were confirmed based on the literature.

In order to further verify these results, a novel miRNA over-representation analysis tool miEAA 2.0^34^ were employed to analyze the top 50 predicted miRNAs of breast neoplasms and leukemia. Supplementary table 12 listed the top 10 analysis results for breast neoplasms, and Supplementary table 13 listed the top 20 analysis results for leukemia, respectively. Breast neoplasms ranks fifth in supplementary table 12 with the p-value of 4.02 × 10^–14^ and leukemia ranks 17th in supplementary table 13 with the p-value of 9.09 × 10^–15^. There is 44/50 predicted miRNA associate with breast neoplasms and 23/50 miRNA associate with leukemia. This result that some of disease-related predicted miRNAs have also been verified by miEAA 2.0, indicates the reliability of the prediction results.

## Discussion

As the rapid accumulation of newly discovered miRNAs, more and more evidences indicate that miRNAs are closely related to the occurrence and development of various human diseases. Using a computational method to identify the miRNA-disease associations is not only able to reduce time and experiment consumption, but also accelerate drug research and development. In this research, we used cross validation to evaluate the prediction performance by constructing a series of data sets. Case studies are also conducted by applying Seq-SymRF on several important human diseases and miRNAs. For breast neoplasms, leukemia and hsa-mir-21, 66%, 72%, 64% of the predicted miRNA-disease associations in the top 50 are confirmed by database and literature, respectively. The outstanding performance of our model dependent on four reasons: (1) we constructed the data set based on the HMDD which collects the newest experiment confirmed miRNA-disease associations. (2) Instead of selecting the negative sample randomly, we calculate the Euclidean distance between the un-labeled sample and the positive cluster center and select the reliable negative sample. (3) The miRNA sequence features and disease symptom descriptors are used to characterize miRNA and disease, respectively. (4) The random forest classifier is a powerful model and able to complete the binary classification task very easily and efficiently. Of course, our method also has some limitations. For example, after integrating HMDD and symptom-disease network, only a part of diseases in HMDD are selected. Similarly, just a part of miRNAs are collected. The incompleteness of miRNAs and diseases affects the performance of the model. After solving the limitation and integrating more information, we believe that the performance of Seq-SymRF model can be further improved.

## Conclusion

The miRNA sequence and disease symptom play a significant role. Treating them as the feature to train the random forest model is a relatively new approach. Compare to traditional methods, it contains more biological and medical information. We implement the Seq-SymRF model to infer the miRNA-disease associations based on the two features and achieve an excellent performance. From different experimental results, it can be seen that the Seq-SymRF could be a helpful and reliable model to explore the complex relationship between miRNAs and diseases.

## Supplementary information

Supplementary Information.

## Data Availability

All data generated or analysed during this study are included in this published article and its Supplementary Information files. The source code and data of Seq-SymRF is available at https://github.com/LeeKamlong/Seq-SymRF.
