# Supplementary material for: Seq-SymRF: a random forest model predicts potential miRNA-disease associations based on information of sequences and clinical symptoms
Source: Sci Rep. 2020 Oct 21;10:17901. doi: 10.1038/s41598-020-75005-9 (PMC7578641; doi:10.1038/s41598-020-75005-9)
Supplement: Supplementary file 1 — Supplementary Information. [file 41598_2020_75005_MOESM1_ESM.docx]

**Supplementary information**

**Seq-SymRF: A random forest model predicts potential miRNA-disease associations based on information of sequences and clinical symptoms**

Jinlong Li, Xingyu Chen, Qixing Huang, Yang Wang, Yun Xie, Zong Dai, Xiaoyong Zou^*^ and Zhanchao Li^*^

*Correspondence to:

Zhanchao Li^*^

zhanchao8052@gdpu.edu.cn

Xiaoyong Zou^*^

ceszxy@mail.sysu.edu.cn

**Supplementray table:**

**Supplementary table 1** The Acc(%) of different model parameter

|  | | Number of predictor | | | | | |
| --- | --- | --- | --- | --- | --- | --- | --- |
|  |  | 2 | 4 | 8 | 16 | 22 | 32 |
| Number  of  Trees | 100 | 85.61 | 88.37 | 90.58 | 91.98 | 92.34 | 92.68 |
|  | 200 | 85.95 | 88.34 | 90.65 | 92.23 | 92.44 | 92.80 |
|  | 300 | 85.92 | 88.32 | 90.67 | 92.27 | 92.54 | 92.88 |
|  | 400 | 85.92 | 88.37 | 90.69 | 92.21 | 92.48 | 92.80 |
|  | 500 | 86.03 | 88.30 | 90.60 | 92.16 | 92.64 | 92.86 |
|  | 600 | 85.92 | 88.30 | 90.67 | 92.26 | 92.60 | 92.83 |
|  | 700 | 85.84 | 88.28 | 90.65 | 92.26 | 92.52 | 92.76 |
|  | 800 | 85.90 | 88.28 | 90.59 | 92.33 | 92.51 | 92.76 |
|  | 900 | 85.93 | 88.30 | 90.53 | 92.25 | 92.60 | 92.85 |
|  | 1000 | 85.89 | 88.31 | 90.71 | 92.28 | 92.55 | 92.82 |

**Supplementary table 2** The Sen(%) of different model parameter

|  | | Number of predictor | | | | | |
| --- | --- | --- | --- | --- | --- | --- | --- |
|  |  | 2 | 4 | 8 | 16 | 22 | 32 |
| Number  of  Trees | 100 | 85.08 | 89.27 | 90.68 | 91.50 | 91.72 | 91.77 |
|  | 200 | 85.58 | 89.08 | 90.64 | 91.56 | 91.81 | 92.02 |
|  | 300 | 85.68 | 89.07 | 90.81 | 91.71 | 91.98 | 91.86 |
|  | 400 | 85.63 | 89.07 | 90.77 | 91.58 | 91.81 | 91.89 |
|  | 500 | 85.69 | 88.97 | 90.64 | 91.54 | 91.93 | 92.00 |
|  | 600 | 85.51 | 89.20 | 90.72 | 91.79 | 91.95 | 91.98 |
|  | 700 | 85.55 | 89.15 | 90.68 | 91.77 | 91.81 | 91.81 |
|  | 800 | 85.49 | 89.01 | 90.61 | 91.76 | 91.79 | 91.84 |
|  | 900 | 85.61 | 89.09 | 90.60 | 91.72 | 91.88 | 92.01 |
|  | 1000 | 85.54 | 89.09 | 90.68 | 91.71 | 91.87 | 91.90 |

**Supplementary table 3** The Spe(%) of different model parameter

|  | | Number of predictor | | | | | |
| --- | --- | --- | --- | --- | --- | --- | --- |
|  |  | 2 | 4 | 8 | 16 | 22 | 32 |
| Number  of  Trees | 100 | 86.13 | 87.48 | 90.49 | 92.46 | 92.95 | 93.60 |
|  | 200 | 86.32 | 87.59 | 90.65 | 92.89 | 93.08 | 93.57 |
|  | 300 | 86.15 | 87.58 | 90.53 | 92.82 | 93.10 | 93.90 |
|  | 400 | 86.21 | 87.66 | 90.61 | 92.85 | 93.16 | 93.71 |
|  | 500 | 86.38 | 87.62 | 90.56 | 92.78 | 93.34 | 93.72 |
|  | 600 | 86.32 | 87.39 | 90.62 | 92.73 | 93.25 | 93.68 |
|  | 700 | 86.12 | 87.42 | 90.61 | 92.75 | 93.24 | 93.71 |
|  | 800 | 86.32 | 87.54 | 90.57 | 92.89 | 93.23 | 93.68 |
|  | 900 | 86.26 | 87.51 | 90.46 | 92.78 | 93.30 | 93.70 |
|  | 1000 | 86.24 | 87.53 | 90.73 | 92.85 | 93.22 | 93.74 |

**Supplementary table 4** The Pre(%) of different model parameter

|  | | Number of predictor | | | | | |
| --- | --- | --- | --- | --- | --- | --- | --- |
|  |  | 2 | 4 | 8 | 16 | 22 | 32 |
| Number  of  Trees | 100 | 85.97 | 87.69 | 90.51 | 92.39 | 92.86 | 93.48 |
|  | 200 | 86.21 | 87.77 | 90.65 | 92.79 | 92.99 | 93.47 |
|  | 300 | 86.07 | 87.76 | 90.56 | 92.74 | 93.03 | 93.77 |
|  | 400 | 86.11 | 87.83 | 90.62 | 92.76 | 93.06 | 93.60 |
|  | 500 | 86.27 | 87.78 | 90.56 | 92.69 | 93.25 | 93.61 |
|  | 600 | 86.20 | 87.61 | 90.63 | 92.66 | 93.16 | 93.57 |
|  | 700 | 86.03 | 87.63 | 90.62 | 92.69 | 93.14 | 93.59 |
|  | 800 | 86.19 | 87.71 | 90.57 | 92.81 | 93.13 | 93.56 |
|  | 900 | 86.15 | 87.70 | 90.48 | 92.71 | 93.21 | 93.59 |
|  | 1000 | 86.13 | 87.71 | 90.73 | 92.77 | 93.13 | 93.62 |

**Supplementary table 5** The Mcc of different model parameter

|  | | Number of predictor | | | | | |
| --- | --- | --- | --- | --- | --- | --- | --- |
|  |  | 2 | 4 | 8 | 16 | 22 | 32 |
| Number  of  Trees | 100 | 0.7122 | 0.7675 | 0.8117 | 0.8396 | 0.8468 | 0.8538 |
|  | 200 | 0.7190 | 0.7668 | 0.8129 | 0.8446 | 0.8489 | 0.8560 |
|  | 300 | 0.7183 | 0.7666 | 0.8134 | 0.8454 | 0.8509 | 0.8577 |
|  | 400 | 0.7183 | 0.7674 | 0.8138 | 0.8443 | 0.8497 | 0.8561 |
|  | 500 | 0.7206 | 0.7660 | 0.8119 | 0.8433 | 0.8528 | 0.8574 |
|  | 600 | 0.7184 | 0.7660 | 0.8134 | 0.8453 | 0.8521 | 0.8567 |
|  | 700 | 0.7167 | 0.7658 | 0.8129 | 0.8452 | 0.8505 | 0.8553 |
|  | 800 | 0.7181 | 0.7656 | 0.8118 | 0.8466 | 0.8503 | 0.8554 |
|  | 900 | 0.7186 | 0.7662 | 0.8106 | 0.8451 | 0.8520 | 0.8571 |
|  | 1000 | 0.7178 | 0.7663 | 0.8141 | 0.8457 | 0.8510 | 0.8565 |

**Supplementary table 6** The AUROC of different model parameter

|  | | Number of predictor | | | | | |
| --- | --- | --- | --- | --- | --- | --- | --- |
|  |  | 2 | 4 | 8 | 16 | 22 | 32 |
| Number  of  Trees | 100 | 0.9418 | 0.9578 | 0.9721 | 0.9808 | 0.9819 | 0.9825 |
|  | 200 | 0.9382 | 0.9582 | 0.9726 | 0.9806 | 0.9818 | 0.9829 |
|  | 300 | 0.9407 | 0.9592 | 0.9724 | 0.9806 | 0.9820 | 0.9832 |
|  | 400 | 0.9405 | 0.9589 | 0.9738 | 0.9809 | 0.9820 | 0.9833 |
|  | 500 | 0.9403 | 0.9588 | 0.9720 | 0.9803 | 0.9826 | 0.9835 |
|  | 600 | 0.9412 | 0.9586 | 0.9728 | 0.9804 | 0.9825 | 0.9832 |
|  | 700 | 0.9413 | 0.9587 | 0.9728 | 0.9801 | 0.9823 | 0.9833 |
|  | 800 | 0.9385 | 0.9592 | 0.9726 | 0.9809 | 0.9822 | 0.9833 |
|  | 900 | 0.9410 | 0.9586 | 0.9721 | 0.9802 | 0.9823 | 0.9834 |
|  | 1000 | 0.9400 | 0.9587 | 0.9730 | 0.9804 | 0.9821 | 0.9835 |

**Supplementary table 7** The AUPRC of different model parameter

|  | | Number of predictor | | | | | |
| --- | --- | --- | --- | --- | --- | --- | --- |
|  |  | 2 | 4 | 8 | 16 | 22 | 32 |
| Number  of  Trees | 100 | 0.9397 | 0.9567 | 0.9737 | 0.9833 | 0.9846 | 0.9856 |
|  | 200 | 0.9345 | 0.9579 | 0.9743 | 0.9834 | 0.9846 | 0.9858 |
|  | 300 | 0.9371 | 0.9594 | 0.9739 | 0.9833 | 0.9846 | 0.9859 |
|  | 400 | 0.9371 | 0.9586 | 0.9756 | 0.9835 | 0.9847 | 0.9861 |
|  | 500 | 0.9374 | 0.9585 | 0.9736 | 0.9830 | 0.9852 | 0.9862 |
|  | 600 | 0.9380 | 0.9583 | 0.9747 | 0.9831 | 0.9851 | 0.9860 |
|  | 700 | 0.9386 | 0.9582 | 0.9746 | 0.9828 | 0.9851 | 0.9860 |
|  | 800 | 0.9355 | 0.9585 | 0.9743 | 0.9835 | 0.9849 | 0.9860 |
|  | 900 | 0.9378 | 0.9585 | 0.9737 | 0.9828 | 0.9850 | 0.9862 |
|  | 1000 | 0.9365 | 0.9581 | 0.9747 | 0.9831 | 0.9848 | 0.9863 |

**Supplementary table 8** The description of the verifying for prediction capability for potential miRNA-disease associations.

| **Algorithm 1 Prediction capability for potential miRNA-disease associations** |
| --- |
| **Input:** Threshold T Positive sample P Reliable negative sample RN Size of positive sample *np*  Size of negative sample *nn*  **Output:** Non-redundant positive sample NRP Non-redundant negative sample NRN Size of non-redundant positive sample *k* Size of non-redundant negative sample *l*  1:for i = 1: *np*  2:randomly select a from P  3:calculate mDPS(a,P)  4:if max((mDPS(a,P)) < T then  5:a∈NRP  6:end for  7:for j = 1: *nn*  8:randomly select b from RN  9:calculate mDPS(b,NRP) and mDPS(b,RN)  10:if max(mDPS(b,NRP)) < T & max(mDPS(b,RN)) < T  11:b∈NRN  12:when *l* = *k* end for  13:combine NRP and NRN |

**Supplementary table 9** Prediction list of the top 50 prioritized miRNAs associated with breast neoplasms

| miRNA | Score | Evidence | miRNA | Score | Evidence |
| --- | --- | --- | --- | --- | --- |
| hsa-mir-21 | 0.98 | dbDEMC | hsa-mir-409 | 0.96 | unconfirmed |
| hsa-mir-27b | 0.98 | dbDEMC | hsa-mir-410 | 0.96 | unconfirmed |
| hsa-mir-130a | 0.98 | dbDEMC | hsa-mir-146b | 0.96 | unconfirmed |
| hsa-mir-30e | 0.98 | unconfirmed | hsa-mir-520c | 0.96 | unconfirmed |
| hsa-mir-208a | 0.97 | dbDEMC | hsa-mir-524 | 0.96 | unconfirmed |
| hsa-mir-34a | 0.97 | dbDEMC | hsa-mir-518d | 0.96 | unconfirmed |
| hsa-mir-302a | 0.97 | dbDEMC | hsa-mir-505 | 0.96 | dbDEMC |
| hsa-mir-20a | 0.96 | dbDEMC | hsa-mir-3162 | 0.96 | unconfirmed |
| hsa-mir-22 | 0.96 | dbDEMC | hsa-mir-3170 | 0.96 | dbDEMC |
| hsa-mir-23a | 0.96 | dbDEMC | hsa-mir-4756 | 0.96 | unconfirmed |
| hsa-mir-31 | 0.96 | dbDEMC | hsa-let-7c | 0.95 | dbDEMC |
| hsa-mir-95 | 0.96 | dbDEMC | hsa-let-7f-1 | 0.95 | unconfirmed |
| hsa-mir-107 | 0.96 | dbDEMC | hsa-mir-19a | 0.95 | dbDEMC |
| hsa-let-7g | 0.96 | dbDEMC | hsa-mir-26a-1 | 0.95 | unconfirmed |
| hsa-mir-122 | 0.96 | dbDEMC | hsa-mir-26b | 0.95 | dbDEMC |
| hsa-mir-125b-1 | 0.96 | unconfirmed | hsa-mir-27a | 0.95 | dbDEMC |
| hsa-mir-153-2 | 0.96 | unconfirmed | hsa-mir-101-1 | 0.95 | unconfirmed |
| hsa-mir-125a | 0.96 | dbDEMC | hsa-mir-148a | 0.95 | dbDEMC |
| hsa-mir-125b-2 | 0.96 | unconfirmed | hsa-mir-224 | 0.95 | dbDEMC |
| hsa-mir-129-2 | 0.96 | unconfirmed | hsa-mir-23b | 0.95 | dbDEMC |
| hsa-mir-195 | 0.96 | dbDEMC | hsa-mir-142 | 0.95 | unconfirmed |
| hsa-mir-206 | 0.96 | dbDEMC | hsa-mir-145 | 0.95 | dbDEMC |
| hsa-mir-155 | 0.96 | dbDEMC | hsa-mir-126 | 0.95 | dbDEMC |
| hsa-mir-34c | 0.96 | dbDEMC | hsa-mir-146a | 0.95 | dbDEMC |
| hsa-mir-302d | 0.96 | dbDEMC | hsa-mir-184 | 0.95 | dbDEMC |

**Supplementary table 10** Prediction list of the top 50 prioritized miRNAs associated with leukemia

| miRNA | Score | Evidence | miRNA | Score | Evidence |
| --- | --- | --- | --- | --- | --- |
| hsa-mir-21 | 0.92 | dbDEMC | hsa-mir-489 | 0.88 | unconfirmed |
| hsa-mir-146a | 0.92 | dbDEMC | hsa-mir-505 | 0.88 | dbDEMC |
| hsa-mir-142 | 0.91 | dbDEMC | hsa-mir-449c | 0.88 | unconfirmed |
| hsa-let-7a-1 | 0.91 | unconfirmed | hsa-mir-26b | 0.88 | dbDEMC |
| hsa-mir-19a | 0.90 | dbDEMC | hsa-mir-106a | 0.88 | dbDEMC |
| hsa-mir-20a | 0.90 | dbDEMC | hsa-mir-16-1 | 0.87 | unconfirmed |
| hsa-mir-494 | 0.90 | dbDEMC | hsa-mir-19b-1 | 0.87 | dbDEMC |
| hsa-let-7b | 0.89 | dbDEMC | hsa-mir-31 | 0.87 | dbDEMC |
| hsa-mir-17 | 0.89 | dbDEMC | hsa-mir-101-1 | 0.87 | unconfirmed |
| hsa-mir-22 | 0.89 | dbDEMC | hsa-mir-511 | 0.87 | unconfirmed |
| hsa-mir-26a-1 | 0.89 | unconfirmed | hsa-mir-23b | 0.87 | dbDEMC |
| hsa-mir-145 | 0.89 | dbDEMC | hsa-let-7c | 0.86 | dbDEMC |
| hsa-mir-125b-2 | 0.89 | unconfirmed | hsa-mir-199a-1 | 0.86 | dbDEMC |
| hsa-mir-30c-1 | 0.89 | dbDEMC | hsa-mir-125b-1 | 0.86 | dbDEMC |
| hsa-mir-26a-2 | 0.89 | dbDEMC | hsa-mir-449a | 0.86 | unconfirmed |
| hsa-mir-93 | 0.89 | dbDEMC | hsa-let-7a-3 | 0.85 | unconfirmed |
| hsa-mir-194-1 | 0.89 | unconfirmed | hsa-mir-15a | 0.85 | dbDEMC |
| hsa-let-7f-1 | 0.88 | dbDEMC | hsa-mir-32 | 0.85 | dbDEMC |
| hsa-mir-92a-2 | 0.88 | unconfirmed | hsa-mir-153-2 | 0.85 | unconfirmed |
| hsa-mir-224 | 0.88 | dbDEMC | hsa-mir-125a | 0.85 | dbDEMC |
| hsa-mir-122 | 0.88 | unconfirmed | hsa-mir-195 | 0.85 | dbDEMC |
| hsa-mir-126 | 0.88 | dbDEMC | hsa-mir-206 | 0.85 | dbDEMC |
| hsa-mir-150 | 0.88 | dbDEMC | hsa-mir-106b | 0.85 | dbDEMC |
| hsa-mir-29c | 0.88 | dbDEMC | hsa-mir-34b | 0.85 | dbDEMC |
| hsa-mir-130b | 0.88 | dbDEMC | hsa-mir-34c | 0.85 | dbDEMC |

**Supplementary table 11** Prediction list of the top 50 prioritized disease associated with hsa-mir-21

| disease | Score | | Evidence | disease | Score | Evidence |
| --- | --- | --- | --- | --- | --- | --- |
| Coronary Artery Disease | 0.99 | PMID: 25656948 | | Epilepsy | 0.95 | PMID: 24560344 |
| Glioma | 0.98 | PMID: 26701969 | | Colitis, Ulcerative | 0.95 | PMID: 30530166 |
| Nephrotic Syndrome | 0.98 | PMID: 24992527 | | Lichen Planus | 0.95 | PMID: 29480379 |
| Odontogenic Tumors | 0.98 | unconfirmed | | Cleidocranial Dysplasia | 0.95 | unconfirmed |
| Cataract | 0.98 | PMID: 27586871 | | Silicosis | 0.95 | PMID: 26784015 |
| Hyperglycemia | 0.98 | PMID: 28627440 | | Leiomyoma | 0.95 | PMID: 29747655 |
| Lymphoma, Non-Hodgkin | 0.98 | PMID: 29067124 | | Lyme Disease | 0.95 | unconfirmed |
| Retinoblastoma | 0.97 | PMID: 27600360 | | Guillain-Barre Syndrome | 0.95 | unconfirmed |
| Lymphoma | 0.97 | unconfirmed | | Muscular Dystrophy, Duchenne | 0.94 | PMID: 30692507 |
| Lymphoma, T-Cell | 0.97 | PMID: 27329723 | | Mycosis Fungoides | 0.94 | PMID: 25503151 |
| Hyperuricemia | 0.97 | unconfirmed | | Alopecia | 0.94 | unconfirmed |
| Osteonecrosis | 0.97 | PMID: 30588193 | | Diabetes Mellitus, Type 1 | 0.94 | PMID: 24937532 |
| Pelvic Organ Prolapse | 0.97 | unconfirmed | | Carcinoma, Embryonal | 0.94 | PMID: 24977712 |
| Carcinoma, Renal Cell | 0.96 | PMID: 27814278 | | Nevus | 0.94 | unconfirmed |
| Leukemia, Promyelocytic, Acute | 0.96 | PMID: 20143188 | | Aortic Aneurysm | 0.94 | PMID: 29519942 |
| Hirschsprung Disease | 0.96 | unconfirmed | | Leukemia, Myeloid, Chronic-Phase | 0.94 | PMID: 25575817 |
| Chondrosarcoma | 0.96 | unconfirmed | | Prader-Willi Syndrome | 0.94 | unconfirmed |
| Huntington Disease | 0.96 | unconfirmed | | Digeorge Syndrome | 0.94 | PMID: 26867589 |
| Biliary Atresia | 0.96 | PMID: 26927196 | | Anemia | 0.94 | PMID: 30657053 |
| Fatty Liver, Alcoholic | 0.96 | unconfirmed | | Osteosarcoma | 0.93 | PMID: 28742209 |
| Diabetic Cardiomyopathies | 0.96 | PMID: 30180843 | | Multiple Sclerosis | 0.93 | PMID: 30698680 |
| Hearing Loss | 0.96 | PMID: 28224282 | | Medulloblastoma | 0.93 | PMID: 29280516 |
| Hypogonadism | 0.96 | unconfirmed | | Choriocarcinoma | 0.93 | PMID: 27922982 |
| Ganglioglioma | 0.96 | unconfirmed | | Down Syndrome | 0.93 | unconfirmed |
| Pheochromocytoma | 0.96 | unconfirmed | | Coronavirus Infections | 0.93 | unconfirmed |

**Supplement table 12 The top 10 over-representation analysis results of the top 50 predicted miRNA relation to breast neoplasms**

| **Category** | **Subcategory** | **Enrichment** | **P-value** | **P-adjusted** | **Q-value** | **Expected** | **Observed** | **miRNAs/precursors** |
| --- | --- | --- | --- | --- | --- | --- | --- | --- |
| Diseases (HMDD) | Pancreatic Neoplasms | over-represented | 1.00e-`17 | 6.58e-15 | 6.58e-15 | 9.80603 | 37 | hsa-mir-21; hsa-mir-27b; hsa-mir-130a; hsa-mir-208a; hsa-mir-34a; hsa-mir-20a; hsa-mir-22; hsa-mir-23a; hsa-mir-31; hsa-mir-95; hsa-mir-107; hsa-let-7g; hsa-mir-122; hsa-mir-125b-1; hsa-mir-125a; hsa-mir-125b-2; hsa-mir-195; hsa-mir-206; hsa-mir-155; hsa-mir-34c; hsa-mir-410; hsa-mir-146b; hsa-mir-505; hsa-let-7c; hsa-let-7f-1; hsa-mir-19a; hsa-mir-26a-1; hsa-mir-26b; hsa-mir-27a; hsa-mir-101-1; hsa-mir-148a; hsa-mir-224; hsa-mir-23b; hsa-mir-142; hsa-mir-145; hsa-mir-126; hsa-mir-146a |
| Diseases (HMDD) | Gastric Neoplasms | over-represented | 6.70e-17 | 2.20e-14 | 2.20e-14 | 16.2177 | 44 | hsa-mir-21; hsa-mir-27b; hsa-mir-130a; hsa-mir-30e; hsa-mir-208a; hsa-mir-34a; hsa-mir-302a; hsa-mir-20a; hsa-mir-22; hsa-mir-23a; hsa-mir-31; hsa-mir-95; hsa-mir-107; hsa-let-7g; hsa-mir-122; hsa-mir-125b-1; hsa-mir-125a; hsa-mir-125b-2; hsa-mir-129-2; hsa-mir-195; hsa-mir-206; hsa-mir-155; hsa-mir-34c; hsa-mir-302d; hsa-mir-409; hsa-mir-410; hsa-mir-146b; hsa-mir-520c; hsa-mir-524; hsa-mir-505; hsa-let-7f-1; hsa-mir-19a; hsa-mir-26a-1; hsa-mir-26b; hsa-mir-27a; hsa-mir-101-1; hsa-mir-148a; hsa-mir-224; hsa-mir-23b; hsa-mir-142; hsa-mir-145; hsa-mir-126; hsa-mir-146a; hsa-mir-184 |
| Diseases (HMDD) | Glioblastoma | over-represented | 2.42e-16 | 5.29e-14 | 5.29e-14 | 10.6681 | 37 | hsa-mir-21; hsa-mir-27b; hsa-mir-130a; hsa-mir-30e; hsa-mir-208a; hsa-mir-34a; hsa-mir-302a; hsa-mir-20a; hsa-mir-22; hsa-mir-31; hsa-mir-95; hsa-let-7g; hsa-mir-125b-1; hsa-mir-153-2; hsa-mir-125a; hsa-mir-125b-2; hsa-mir-195; hsa-mir-206; hsa-mir-155; hsa-mir-34c; hsa-mir-302d; hsa-mir-146b; hsa-mir-524; hsa-mir-505; hsa-let-7c; hsa-mir-19a; hsa-mir-26a-1; hsa-mir-27a; hsa-mir-101-1; hsa-mir-148a; hsa-mir-224; hsa-mir-23b; hsa-mir-142; hsa-mir-145; hsa-mir-126; hsa-mir-146a; hsa-mir-184 |
| Diseases (HMDD) | Ovarian Neoplasms | over-represented | 4.32e-15 | 7.08e-13 | 7.08e-13 | 11.5302 | 37 | hsa-mir-21; hsa-mir-27b; hsa-mir-130a; hsa-mir-30e; hsa-mir-34a; hsa-mir-302a; hsa-mir-20a; hsa-mir-22; hsa-mir-23a; hsa-mir-31; hsa-mir-107; hsa-let-7g; hsa-mir-125b-1; hsa-mir-153-2; hsa-mir-125a; hsa-mir-125b-2; hsa-mir-129-2; hsa-mir-195; hsa-mir-206; hsa-mir-155; hsa-mir-34c; hsa-mir-302d; hsa-mir-409; hsa-mir-146b; hsa-let-7c; hsa-let-7f-1; hsa-mir-19a; hsa-mir-27a; hsa-mir-101-1; hsa-mir-148a; hsa-mir-224; hsa-mir-23b; hsa-mir-142; hsa-mir-145; hsa-mir-126; hsa-mir-146a; hsa-mir-184 |
| Diseases (HMDD) | Breast Neoplasms | over-represented | 4.02e-14 | 5.28e-12 | 5.28e-12 | 18.75 | 44 | hsa-mir-21; hsa-mir-27b; hsa-mir-130a; hsa-mir-30e; hsa-mir-208a; hsa-mir-34a; hsa-mir-302a; hsa-mir-20a; hsa-mir-22; hsa-mir-23a; hsa-mir-31; hsa-mir-107; hsa-let-7g; hsa-mir-122; hsa-mir-125b-1; hsa-mir-153-2; hsa-mir-125a; hsa-mir-125b-2; hsa-mir-129-2; hsa-mir-195; hsa-mir-206; hsa-mir-155; hsa-mir-34c; hsa-mir-302d; hsa-mir-409; hsa-mir-410; hsa-mir-146b; hsa-mir-520c; hsa-mir-505; hsa-let-7c; hsa-let-7f-1; hsa-mir-19a; hsa-mir-26a-1; hsa-mir-26b; hsa-mir-27a; hsa-mir-101-1; hsa-mir-148a; hsa-mir-224; hsa-mir-23b; hsa-mir-142; hsa-mir-145; hsa-mir-126; hsa-mir-146a; hsa-mir-184 |
| Diseases (HMDD) | Atherosclerosis | over-represented | 7.90e-14 | 8.64e-12 | 8.64e-12 | 5.01078 | 25 | hsa-mir-21; hsa-mir-27b; hsa-mir-30e; hsa-mir-34a; hsa-mir-302a; hsa-mir-20a; hsa-mir-22; hsa-mir-23a; hsa-mir-31; hsa-mir-107; hsa-let-7g; hsa-mir-122; hsa-mir-125a; hsa-mir-206; hsa-mir-155; hsa-mir-410; hsa-mir-146b; hsa-let-7c; hsa-mir-19a; hsa-mir-26b; hsa-mir-23b; hsa-mir-142; hsa-mir-145; hsa-mir-126; hsa-mir-146a |
| Diseases (HMDD) | Glioma | over-represented | 2.10e-13 | 1.69e-11 | 1.69e-11 | 10.5603 | 34 | hsa-mir-21; hsa-mir-27b; hsa-mir-130a; hsa-mir-30e; hsa-mir-34a; hsa-mir-302a; hsa-mir-20a; hsa-mir-23a; hsa-mir-31; hsa-mir-95; hsa-mir-107; hsa-let-7g; hsa-mir-122; hsa-mir-125b-1; hsa-mir-125a; hsa-mir-125b-2; hsa-mir-129-2; hsa-mir-195; hsa-mir-155; hsa-mir-302d; hsa-mir-410; hsa-mir-146b; hsa-mir-520c; hsa-mir-524; hsa-mir-19a; hsa-mir-26b; hsa-mir-27a; hsa-mir-148a; hsa-mir-224; hsa-mir-23b; hsa-mir-142; hsa-mir-145; hsa-mir-126; hsa-mir-184 |
| Diseases (HMDD) | Leukemia, Myeloid, Acute | over-represented | 2.31e-13 | 1.69e-11 | 1.69e-11 | 6.78879 | 28 | hsa-mir-21; hsa-mir-27b; hsa-mir-130a; hsa-mir-34a; hsa-mir-20a; hsa-mir-22; hsa-mir-23a; hsa-let-7g; hsa-mir-122; hsa-mir-125b-1; hsa-mir-125a; hsa-mir-195; hsa-mir-155; hsa-mir-34c; hsa-mir-409; hsa-mir-146b; hsa-let-7c; hsa-let-7f-1; hsa-mir-19a; hsa-mir-26a-1; hsa-mir-27a; hsa-mir-148a; hsa-mir-224; hsa-mir-23b; hsa-mir-142; hsa-mir-145; hsa-mir-126; hsa-mir-146a |
| Diseases (HMDD) | Melanoma | over-represented | 2.25e-13 | 1.69e-11 | 1.69e-11 | 12.069 | 36 | hsa-mir-21; hsa-mir-27b; hsa-mir-30e; hsa-mir-34a; hsa-mir-302a; hsa-mir-20a; hsa-mir-22; hsa-mir-23a; hsa-mir-31; hsa-mir-107; hsa-let-7g; hsa-mir-122; hsa-mir-125b-1; hsa-mir-153-2; hsa-mir-125a; hsa-mir-125b-2; hsa-mir-195; hsa-mir-206; hsa-mir-155; hsa-mir-34c; hsa-mir-302d; hsa-mir-146b; hsa-mir-520c; hsa-mir-524; hsa-let-7c; hsa-let-7f-1; hsa-mir-19a; hsa-mir-26a-1; hsa-mir-26b; hsa-mir-148a; hsa-mir-224; hsa-mir-23b; hsa-mir-142; hsa-mir-145; hsa-mir-126; hsa-mir-146a |
| Diseases (HMDD) | Neoplasms unspecific | over-represented | 2.62e-13 | 1.72e-11 | 1.72e-11 | 14.6013 | 39 | hsa-mir-21; hsa-mir-27b; hsa-mir-130a; hsa-mir-30e; hsa-mir-208a; hsa-mir-34a; hsa-mir-302a; hsa-mir-20a; hsa-mir-22; hsa-mir-23a; hsa-mir-31; hsa-mir-107; hsa-let-7g; hsa-mir-122; hsa-mir-125b-1; hsa-mir-153-2; hsa-mir-125a; hsa-mir-125b-2; hsa-mir-195; hsa-mir-206; hsa-mir-155; hsa-mir-34c; hsa-mir-302d; hsa-mir-146b; hsa-mir-520c; hsa-let-7c; hsa-let-7f-1; hsa-mir-19a; hsa-mir-26b; hsa-mir-27a; hsa-mir-101-1; hsa-mir-148a; hsa-mir-224; hsa-mir-23b; hsa-mir-142; hsa-mir-145; hsa-mir-126; hsa-mir-146a; hsa-mir-184 |

**Supplement table 13 The top 20 over-representation analysis results of the top 50 predicted miRNA relation to leukemia**

| **Category** | **Subcategory** | **Enrichment** | **P-value** | **P-adjusted** | **Q-value** | **Expected** | **Observed** | **miRNAs/precursors** |
| --- | --- | --- | --- | --- | --- | --- | --- | --- |
| Diseases (HMDD) | Pancreatic Neoplasms | over-represented | 4.06e-24 | 2.63e-21 | 2.63e-21 | 9.80603 | 42 | hsa-mir-21; hsa-mir-146a; hsa-mir-142; hsa-let-7a-1; hsa-mir-19a; hsa-mir-20a; hsa-mir-494; hsa-let-7b; hsa-mir-17; hsa-mir-22; hsa-mir-26a-1; hsa-mir-145; hsa-mir-125b-2; hsa-mir-30c-1; hsa-mir-26a-2; hsa-mir-194-1; hsa-let-7f-1; hsa-mir-224; hsa-mir-122; hsa-mir-126; hsa-mir-150; hsa-mir-29c; hsa-mir-130b; hsa-mir-505; hsa-mir-26b; hsa-mir-106a; hsa-mir-16-1; hsa-mir-31; hsa-mir-101-1; hsa-mir-23b; hsa-let-7c; hsa-mir-199a-1; hsa-mir-125b-1; hsa-let-7a-3; hsa-mir-15a; hsa-mir-32; hsa-mir-125a; hsa-mir-195; hsa-mir-206; hsa-mir-106b; hsa-mir-34b; hsa-mir-34c |
| Diseases (HMDD) | Carcinoma, Renal Cell | over-represented | 8.91e-20 | 2.46e-17 | 2.46e-17 | 9.32112 | 38 | hsa-mir-21; hsa-mir-146a; hsa-mir-142; hsa-let-7a-1; hsa-mir-19a; hsa-mir-20a; hsa-let-7b; hsa-mir-17; hsa-mir-22; hsa-mir-26a-1; hsa-mir-145; hsa-mir-30c-1; hsa-mir-26a-2; hsa-mir-93; hsa-let-7f-1; hsa-mir-92a-2; hsa-mir-224; hsa-mir-122; hsa-mir-126; hsa-mir-150; hsa-mir-29c; hsa-mir-130b; hsa-mir-489; hsa-mir-26b; hsa-mir-106a; hsa-mir-16-1; hsa-mir-19b-1; hsa-mir-101-1; hsa-mir-23b; hsa-let-7c; hsa-mir-199a-1; hsa-let-7a-3; hsa-mir-15a; hsa-mir-195; hsa-mir-206; hsa-mir-106b; hsa-mir-34b; hsa-mir-34c |
| Diseases (HMDD) | Ovarian Neoplasms | over-represented | 1.14e-19 | 2.46e-17 | 2.46e-17 | 11.5302 | 41 | hsa-mir-21; hsa-mir-146a; hsa-mir-142; hsa-let-7a-1; hsa-mir-19a; hsa-mir-20a; hsa-mir-494; hsa-let-7b; hsa-mir-17; hsa-mir-22; hsa-mir-145; hsa-mir-125b-2; hsa-mir-30c-1; hsa-mir-93; hsa-mir-194-1; hsa-let-7f-1; hsa-mir-92a-2; hsa-mir-224; hsa-mir-126; hsa-mir-150; hsa-mir-29c; hsa-mir-130b; hsa-mir-489; hsa-mir-106a; hsa-mir-16-1; hsa-mir-19b-1; hsa-mir-31; hsa-mir-101-1; hsa-mir-23b; hsa-let-7c; hsa-mir-199a-1; hsa-mir-125b-1; hsa-mir-449a; hsa-let-7a-3; hsa-mir-153-2; hsa-mir-125a; hsa-mir-195; hsa-mir-206; hsa-mir-106b; hsa-mir-34b; hsa-mir-34c |
| Diseases (HMDD) | Breast Neoplasms | over-represented | 2.23e-19 | 3.61e-17 | 3.61e-17 | 18.75 | 48 | hsa-mir-21; hsa-mir-146a; hsa-mir-142; hsa-let-7a-1; hsa-mir-19a; hsa-mir-20a; hsa-mir-494; hsa-let-7b; hsa-mir-17; hsa-mir-22; hsa-mir-26a-1; hsa-mir-145; hsa-mir-125b-2; hsa-mir-30c-1; hsa-mir-26a-2; hsa-mir-93; hsa-mir-194-1; hsa-let-7f-1; hsa-mir-92a-2; hsa-mir-224; hsa-mir-122; hsa-mir-126; hsa-mir-150; hsa-mir-29c; hsa-mir-130b; hsa-mir-489; hsa-mir-505; hsa-mir-26b; hsa-mir-106a; hsa-mir-16-1; hsa-mir-19b-1; hsa-mir-31; hsa-mir-101-1; hsa-mir-23b; hsa-let-7c; hsa-mir-199a-1; hsa-mir-125b-1; hsa-mir-449a; hsa-let-7a-3; hsa-mir-15a; hsa-mir-32; hsa-mir-153-2; hsa-mir-125a; hsa-mir-195; hsa-mir-206; hsa-mir-106b; hsa-mir-34b; hsa-mir-34c |
| Diseases (HMDD) | Glioblastoma | over-represented | 1.25e-18 | 1.62e-16 | 1.62e-16 | 10.6681 | 39 | hsa-mir-21; hsa-mir-146a; hsa-mir-142; hsa-let-7a-1; hsa-mir-19a; hsa-mir-20a; hsa-mir-494; hsa-let-7b; hsa-mir-17; hsa-mir-22; hsa-mir-26a-1; hsa-mir-145; hsa-mir-125b-2; hsa-mir-30c-1; hsa-mir-26a-2; hsa-mir-93; hsa-mir-92a-2; hsa-mir-224; hsa-mir-126; hsa-mir-29c; hsa-mir-130b; hsa-mir-505; hsa-mir-106a; hsa-mir-16-1; hsa-mir-19b-1; hsa-mir-31; hsa-mir-101-1; hsa-mir-23b; hsa-let-7c; hsa-mir-125b-1; hsa-mir-449a; hsa-let-7a-3; hsa-mir-15a; hsa-mir-32; hsa-mir-153-2; hsa-mir-125a; hsa-mir-195; hsa-mir-206; hsa-mir-34c |
| Diseases (HMDD) | Prostate Neoplasms | over-represented | 2.63e-18 | 2.83e-16 | 2.83e-16 | 13.2543 | 42 | hsa-mir-21; hsa-mir-146a; hsa-let-7a-1; hsa-mir-19a; hsa-mir-20a; hsa-mir-494; hsa-let-7b; hsa-mir-17; hsa-mir-22; hsa-mir-26a-1; hsa-mir-145; hsa-mir-125b-2; hsa-mir-30c-1; hsa-mir-26a-2; hsa-mir-93; hsa-mir-194-1; hsa-mir-92a-2; hsa-mir-224; hsa-mir-122; hsa-mir-126; hsa-mir-150; hsa-mir-29c; hsa-mir-130b; hsa-mir-26b; hsa-mir-106a; hsa-mir-16-1; hsa-mir-19b-1; hsa-mir-31; hsa-mir-101-1; hsa-mir-23b; hsa-let-7c; hsa-mir-199a-1; hsa-mir-125b-1; hsa-mir-449a; hsa-mir-15a; hsa-mir-32; hsa-mir-153-2; hsa-mir-195; hsa-mir-206; hsa-mir-106b; hsa-mir-34b; hsa-mir-34c |
| Diseases (HMDD) | Leukemia, Myeloid, Acute | over-represented | 1.05e-17 | 8.62e-16 | 8.62e-16 | 6.78879 | 32 | hsa-mir-21; hsa-mir-146a; hsa-mir-142; hsa-let-7a-1; hsa-mir-19a; hsa-mir-20a; hsa-mir-494; hsa-let-7b; hsa-mir-17; hsa-mir-22; hsa-mir-26a-1; hsa-mir-145; hsa-mir-30c-1; hsa-mir-26a-2; hsa-let-7f-1; hsa-mir-92a-2; hsa-mir-224; hsa-mir-122; hsa-mir-126; hsa-mir-150; hsa-mir-16-1; hsa-mir-19b-1; hsa-mir-511; hsa-mir-23b; hsa-let-7c; hsa-mir-125b-1; hsa-let-7a-3; hsa-mir-15a; hsa-mir-125a; hsa-mir-195; hsa-mir-34b; hsa-mir-34c |
| Diseases (HMDD) | Neoplasms unspecific | over-represented | 1.07e-17 | 8.62e-16 | 8.62e-16 | 14.6013 | 43 | hsa-mir-21; hsa-mir-146a; hsa-mir-142; hsa-let-7a-1; hsa-mir-19a; hsa-mir-20a; hsa-mir-494; hsa-let-7b; hsa-mir-17; hsa-mir-22; hsa-mir-145; hsa-mir-125b-2; hsa-mir-30c-1; hsa-mir-93; hsa-let-7f-1; hsa-mir-92a-2; hsa-mir-224; hsa-mir-122; hsa-mir-126; hsa-mir-150; hsa-mir-29c; hsa-mir-26b; hsa-mir-106a; hsa-mir-16-1; hsa-mir-19b-1; hsa-mir-31; hsa-mir-101-1; hsa-mir-511; hsa-mir-23b; hsa-let-7c; hsa-mir-199a-1; hsa-mir-125b-1; hsa-mir-449a; hsa-let-7a-3; hsa-mir-15a; hsa-mir-32; hsa-mir-153-2; hsa-mir-125a; hsa-mir-195; hsa-mir-206; hsa-mir-106b; hsa-mir-34b; hsa-mir-34c |
| Diseases (HMDD) | Melanoma | over-represented | 1.24e-17 | 8.94e-16 | 8.94e-16 | 12.069 | 40 | hsa-mir-21; hsa-mir-146a; hsa-mir-142; hsa-let-7a-1; hsa-mir-19a; hsa-mir-20a; hsa-let-7b; hsa-mir-17; hsa-mir-22; hsa-mir-26a-1; hsa-mir-145; hsa-mir-125b-2; hsa-mir-30c-1; hsa-mir-26a-2; hsa-mir-93; hsa-mir-194-1; hsa-let-7f-1; hsa-mir-224; hsa-mir-122; hsa-mir-126; hsa-mir-150; hsa-mir-130b; hsa-mir-26b; hsa-mir-106a; hsa-mir-16-1; hsa-mir-19b-1; hsa-mir-31; hsa-mir-23b; hsa-let-7c; hsa-mir-199a-1; hsa-mir-125b-1; hsa-let-7a-3; hsa-mir-15a; hsa-mir-153-2; hsa-mir-125a; hsa-mir-195; hsa-mir-206; hsa-mir-106b; hsa-mir-34b; hsa-mir-34c |
| Diseases (HMDD) | Gastric Neoplasms | over-represented | 6.70e-17 | 4.33e-15 | 4.33e-15 | 16.2177 | 44 | hsa-mir-21; hsa-mir-146a; hsa-mir-142; hsa-let-7a-1; hsa-mir-19a; hsa-mir-20a; hsa-mir-494; hsa-let-7b; hsa-mir-17; hsa-mir-22; hsa-mir-26a-1; hsa-mir-145; hsa-mir-125b-2; hsa-mir-30c-1; hsa-mir-93; hsa-mir-194-1; hsa-let-7f-1; hsa-mir-224; hsa-mir-122; hsa-mir-126; hsa-mir-150; hsa-mir-29c; hsa-mir-130b; hsa-mir-505; hsa-mir-449c; hsa-mir-26b; hsa-mir-106a; hsa-mir-16-1; hsa-mir-19b-1; hsa-mir-31; hsa-mir-101-1; hsa-mir-23b; hsa-mir-199a-1; hsa-mir-125b-1; hsa-mir-449a; hsa-let-7a-3; hsa-mir-15a; hsa-mir-32; hsa-mir-125a; hsa-mir-195; hsa-mir-206; hsa-mir-106b; hsa-mir-34b; hsa-mir-34c |
| Diseases (HMDD) | Colon Neoplasms | over-represented | 5.76e-16 | 3.39e-14 | 3.39e-14 | 7.65086 | 32 | hsa-mir-21; hsa-mir-146a; hsa-mir-142; hsa-let-7a-1; hsa-mir-19a; hsa-mir-20a; hsa-let-7b; hsa-mir-17; hsa-mir-22; hsa-mir-145; hsa-mir-125b-2; hsa-mir-30c-1; hsa-mir-93; hsa-let-7f-1; hsa-mir-224; hsa-mir-126; hsa-mir-150; hsa-mir-106a; hsa-mir-16-1; hsa-mir-19b-1; hsa-mir-31; hsa-mir-101-1; hsa-mir-23b; hsa-let-7c; hsa-mir-125b-1; hsa-mir-449a; hsa-let-7a-3; hsa-mir-15a; hsa-mir-32; hsa-mir-125a; hsa-mir-195; hsa-mir-106b |
| Diseases (HMDD) | Carcinoma, Hepatocellular | over-represented | 1.70e-15 | 9.19e-14 | 9.19e-14 | 21.0129 | 47 | hsa-mir-21; hsa-mir-146a; hsa-mir-142; hsa-let-7a-1; hsa-mir-19a; hsa-mir-20a; hsa-mir-494; hsa-let-7b; hsa-mir-17; hsa-mir-22; hsa-mir-26a-1; hsa-mir-145; hsa-mir-125b-2; hsa-mir-30c-1; hsa-mir-26a-2; hsa-mir-93; hsa-let-7f-1; hsa-mir-92a-2; hsa-mir-224; hsa-mir-122; hsa-mir-126; hsa-mir-150; hsa-mir-29c; hsa-mir-130b; hsa-mir-489; hsa-mir-505; hsa-mir-26b; hsa-mir-106a; hsa-mir-16-1; hsa-mir-19b-1; hsa-mir-31; hsa-mir-101-1; hsa-mir-511; hsa-mir-23b; hsa-let-7c; hsa-mir-199a-1; hsa-mir-125b-1; hsa-mir-449a; hsa-let-7a-3; hsa-mir-15a; hsa-mir-32; hsa-mir-125a; hsa-mir-195; hsa-mir-206; hsa-mir-106b; hsa-mir-34b; hsa-mir-34c |
| Diseases (HMDD) | Inflammation | over-represented | 3.79e-15 | 1.75e-13 | 1.75e-13 | 4.47198 | 25 | hsa-mir-21; hsa-mir-146a; hsa-mir-142; hsa-let-7a-1; hsa-mir-19a; hsa-mir-20a; hsa-let-7b; hsa-mir-17; hsa-mir-22; hsa-mir-145; hsa-mir-93; hsa-let-7f-1; hsa-mir-122; hsa-mir-126; hsa-mir-150; hsa-mir-130b; hsa-mir-449c; hsa-mir-19b-1; hsa-mir-31; hsa-mir-23b; hsa-let-7c; hsa-let-7a-3; hsa-mir-15a; hsa-mir-125a; hsa-mir-206 |
| Diseases (HMDD) | Lung Neoplasms | over-represented | 3.67e-15 | 1.75e-13 | 1.75e-13 | 13.9009 | 40 | hsa-mir-21; hsa-mir-146a; hsa-mir-142; hsa-let-7a-1; hsa-mir-19a; hsa-mir-20a; hsa-mir-494; hsa-let-7b; hsa-mir-17; hsa-mir-22; hsa-mir-26a-1; hsa-mir-145; hsa-mir-30c-1; hsa-mir-26a-2; hsa-mir-93; hsa-let-7f-1; hsa-mir-224; hsa-mir-122; hsa-mir-126; hsa-mir-150; hsa-mir-29c; hsa-mir-130b; hsa-mir-26b; hsa-mir-106a; hsa-mir-19b-1; hsa-mir-31; hsa-mir-101-1; hsa-mir-511; hsa-let-7c; hsa-mir-199a-1; hsa-mir-125b-1; hsa-mir-449a; hsa-let-7a-3; hsa-mir-15a; hsa-mir-32; hsa-mir-125a; hsa-mir-195; hsa-mir-206; hsa-mir-34b; hsa-mir-34c |
| Diseases (HMDD) | Heart Failure | over-represented | 8.65e-15 | 3.68e-13 | 3.68e-13 | 8.94397 | 33 | hsa-mir-21; hsa-mir-146a; hsa-mir-142; hsa-let-7a-1; hsa-mir-19a; hsa-mir-494; hsa-let-7b; hsa-mir-17; hsa-mir-22; hsa-mir-26a-1; hsa-mir-145; hsa-mir-30c-1; hsa-mir-26a-2; hsa-mir-93; hsa-let-7f-1; hsa-mir-92a-2; hsa-mir-122; hsa-mir-126; hsa-mir-150; hsa-mir-130b; hsa-mir-26b; hsa-mir-16-1; hsa-mir-19b-1; hsa-mir-23b; hsa-let-7c; hsa-mir-199a-1; hsa-mir-32; hsa-mir-125a; hsa-mir-195; hsa-mir-206; hsa-mir-106b; hsa-mir-34b; hsa-mir-34c |
| Diseases (HMDD) | Leukemia | over-represented | 9.09e-15 | 3.68e-13 | 3.68e-13 | 3.77155 | 23 | hsa-mir-21; hsa-mir-146a; hsa-mir-142; hsa-mir-19a; hsa-mir-20a; hsa-let-7b; hsa-mir-17; hsa-mir-145; hsa-mir-125b-2; hsa-mir-92a-2; hsa-mir-122; hsa-mir-126; hsa-mir-150; hsa-mir-16-1; hsa-mir-19b-1; hsa-mir-31; hsa-mir-199a-1; hsa-mir-125b-1; hsa-mir-15a; hsa-mir-125a; hsa-mir-195; hsa-mir-34b; hsa-mir-34c |
| Diseases (HMDD) | Colorectal Carcinoma | over-represented | 1.34e-14 | 5.08e-13 | 5.08e-13 | 16.2177 | 42 | hsa-mir-21; hsa-mir-146a; hsa-mir-142; hsa-let-7a-1; hsa-mir-19a; hsa-mir-20a; hsa-let-7b; hsa-mir-17; hsa-mir-22; hsa-mir-26a-1; hsa-mir-145; hsa-mir-125b-2; hsa-mir-26a-2; hsa-mir-93; hsa-mir-194-1; hsa-mir-92a-2; hsa-mir-224; hsa-mir-122; hsa-mir-126; hsa-mir-150; hsa-mir-29c; hsa-mir-130b; hsa-mir-489; hsa-mir-505; hsa-mir-26b; hsa-mir-106a; hsa-mir-16-1; hsa-mir-19b-1; hsa-mir-31; hsa-mir-23b; hsa-let-7c; hsa-mir-199a-1; hsa-mir-125b-1; hsa-let-7a-3; hsa-mir-15a; hsa-mir-32; hsa-mir-125a; hsa-mir-195; hsa-mir-206; hsa-mir-106b; hsa-mir-34b; hsa-mir-34c |
| Diseases (HMDD) | Lymphoma | over-represented | 3.74e-14 | 1.34e-12 | 1.34e-12 | 4.4181 | 24 | hsa-mir-21; hsa-mir-146a; hsa-mir-142; hsa-mir-19a; hsa-mir-20a; hsa-mir-494; hsa-mir-17; hsa-mir-22; hsa-mir-26a-1; hsa-mir-26a-2; hsa-mir-93; hsa-mir-92a-2; hsa-mir-224; hsa-mir-122; hsa-mir-126; hsa-mir-150; hsa-mir-29c; hsa-mir-16-1; hsa-mir-19b-1; hsa-mir-31; hsa-mir-101-1; hsa-mir-23b; hsa-mir-15a; hsa-mir-125a |
| Diseases (HMDD) | Lymphoma, T-Cell | over-represented | 6.80e-14 | 2.31e-12 | 2.31e-12 | 1.67026 | 16 | hsa-mir-21; hsa-mir-146a; hsa-mir-142; hsa-mir-19a; hsa-mir-20a; hsa-mir-17; hsa-mir-26a-1; hsa-mir-125b-2; hsa-mir-26a-2; hsa-mir-93; hsa-mir-92a-2; hsa-mir-26b; hsa-mir-19b-1; hsa-mir-101-1; hsa-mir-511; hsa-mir-125b-1 |
| Diseases (HMDD) | Leukemia, Lymphocytic, Chronic, B-Cell | over-represented | 9.22e-14 | 2.98e-12 | 2.98e-12 | 3.71767 | 22 | hsa-mir-21; hsa-mir-146a; hsa-mir-19a; hsa-mir-20a; hsa-mir-17; hsa-mir-22; hsa-mir-125b-2; hsa-mir-122; hsa-mir-150; hsa-mir-29c; hsa-mir-16-1; hsa-mir-19b-1; hsa-mir-31; hsa-mir-23b; hsa-mir-125b-1; hsa-mir-15a; hsa-mir-125a; hsa-mir-195; hsa-mir-206; hsa-mir-106b; hsa-mir-34b; hsa-mir-34c |

**Supplementary Fig.1 The results of top-50 important features**

**
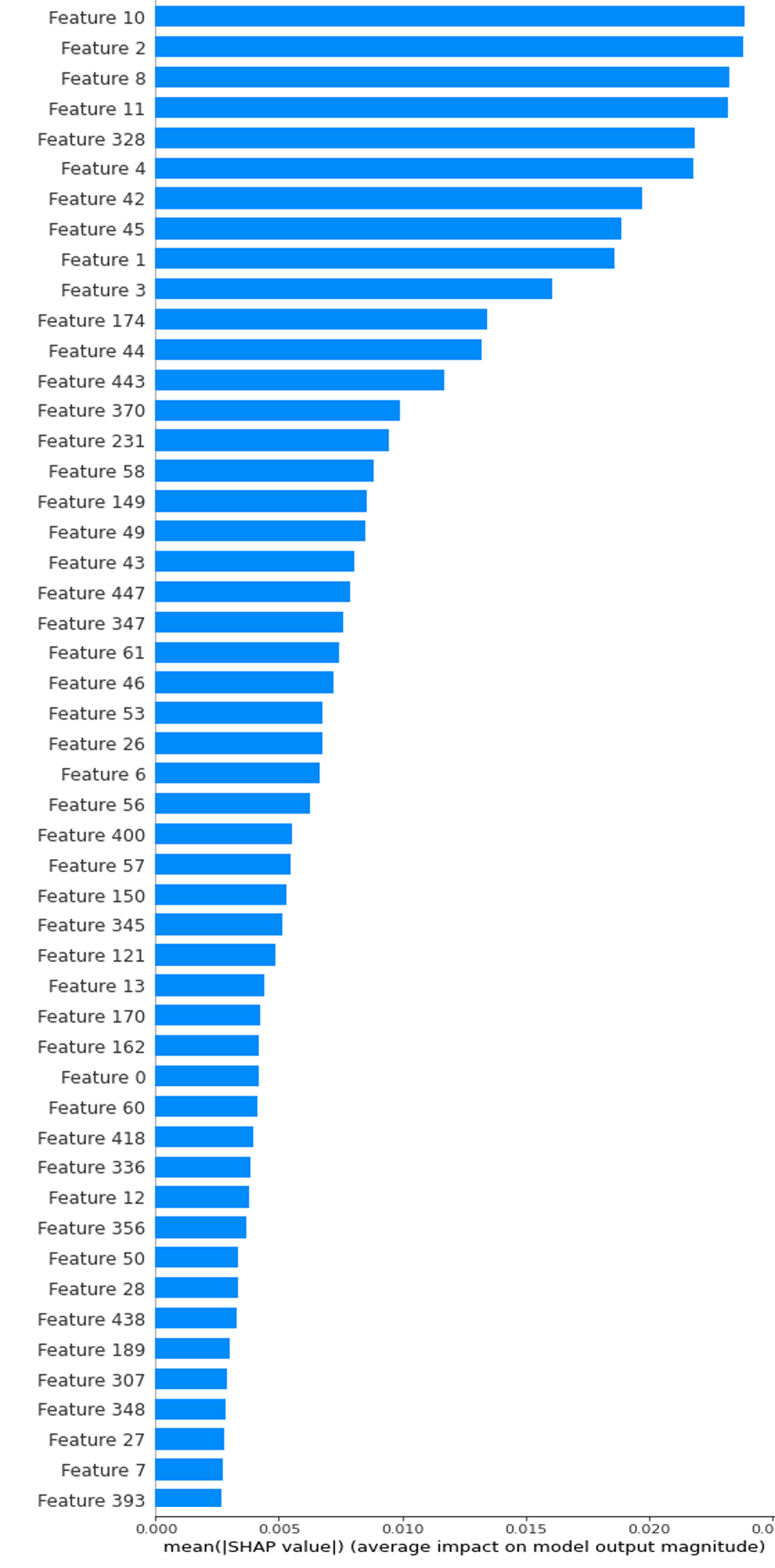
**
